# Does It Matter What Is Said and Who Said It? The Interpretation of Trump’s and Harris’ Statements Among Republican and Democrat Voters

**DOI:** 10.1162/OPMI.a.343

**Published:** 2026-03-23

**Authors:** Nicole Gotzner

**Affiliations:** Cognitive Science Institute, Osnabrück University, Osnabrück, Germany

**Keywords:** pragmatics, implicature, beliefs, cooperation, political language

## Abstract

The Gricean model of communication assumes that cooperation is a precondition for successful communication but humans often use language non-cooperatively. How do language users calibrate cooperation when deciphering communicated content? The current work probes how belief alignment shapes the interpretation of underinformative statements attributed to politicians (Donald Trump or Kamala Harris) presented to self-identified Republican and Democratic participants. The results show that communicated content is more likely to be derived when beliefs align between voter group and speaker. This suggests that we may arrive at different conclusions from the same statement, depending on who the speaker is and how much trust we grant them.

## INTRODUCTION

The influential work of the language philosopher Grice took cooperation to be the premise for any successful communicative exchange (Grice, 1975). Interlocutors should make a contribution “such as is required, at the stage at which it occurs, by the accepted purpose or direction of the talk exchange” (Grice, 1975, page 45). Because of that, human communication is efficient, as speakers can rely on the listener to enrich the meaning of what is said. The Gricean framework of communication has been extended and formalized over the past 50 years and most of the current standard models in pragmatics inherit some version of the Gricean cooperativity principle (relevance theory, Sperber & Wilson, 1986; Rational Speech Act approaches, Frank & Goodman, 2012, see Cummins, 2025 for a recent overview).

However, it is easy to find cases where the premise of cooperation does not hold. Humans use indirect language to manage their social relationships in situations of conflict (Gotzner & Scontras, 2024; Pinker et al., 2008) or even to deceive (Cummins, 2025; Franke et al., 2020; Meibauer, 2005). A prime case of non-cooperative communication is political speech (e.g., Beaver & Stanley, 2023; Lombardi Vallauri, 2016). Politicians routinely have to navigate situations of conflict. When they address us, we have not agreed on a joint goal or direction of the talk exchange. Instead, it is clear that politicians pursue their own strategic goals and that they may address multiple audiences with different beliefs (Henderson & McCready, 2024)

The problem for hearers is that intentions are manifold and they remain hidden. So how do we decipher what the speaker meant? More generally, how do language users calibrate cooperation when content is implicated rather than explicitly stated? The current work probes the role of belief alignment on the interpretations of statements made by opposing politicians, viz. Donald Trump and Kamala Harris, to self-identified Republican and Democratic voters. The results show that communicated content is more likely to be derived when beliefs of hearer and speaker align.

This paper is structured as follows. The first section discusses the background to this research, including the role of cooperation in communication, political speech and the particular type of communicated content tested in this study—scalar implicature. The second part presents the results of an experiment testing the hypothesis that scalar implicatures are more likely to be derived when voter’s beliefs align with that of the political candidate uttering a statement. The final part of the paper presents the conclusions regarding the role of cooperation and social dynamics in communication.

## THEORETICAL AND EMPIRICAL BACKGROUND

### (Non-)Cooperative Language Use

The Gricean model accounts for the efficiency of language and for the fact that interlocutors routinely understand more than what follows from the basic meaning (semantics) of the words that they use. A key feature of human language is that communicative content can be meant or implicated without explicitly being said. For example, a speaker can use the sentence *Mary ate some of the cookies* to mean that ‘Mary ate some but not all of the cookies’ and the hearer routinely derives this so-called scalar implicature. We can show that this implicated meaning is not part of the semantic content of the utterance, as the speaker can cancel the scalar implicature by saying *In fact, she ate all of them*. This cancelability is a common feature of many types of implicature (with the exception of so-called conventional implicatures).

A key challenge to the Gricean assumption about full cooperation as a premise for communication are non-cooperative scenarios. In many situations, an interlocutor’s interests do not coincide exactly but speaker’s interests are best served by misleading (Sperber et al., 2010). When a speaker lies and the hearer recognizes this, the implicated meaning is still understood (Beaver & Stanley, 2023; Franke et al., 2020; Meibauer, 2005). In clearly adversarial situations such as courtroom examinations, implicatures are generated although interlocutors do not cooperate (Asher & Lascarides, 2013). One way to explain this, is that hearers understand the implicated meaning but they do not accept it as true (Sperber et al., 2010). Whether or not the hearer accepts the implicated information, depends on its plausibility and the trust the hearer grants the speaker. More generally, Sperber et al. (2010) argue that interlocutors have to be epistemically vigilant for communication to remain advantageous despite the risk of misinformation. For this purpose, hearers seem to adopt a tentative *stance of trust* (see also Holton, 1994), which may be revoked if the speaker turns out to be uncooperative.

The basic premises of Gricean approaches present a puzzle regarding fundamental insights from evolutionary biology, which suggest that social relationships involve a mixture of cooperation and conflict (e.g., Williams, 2019). Specifically, it is not clear to what extent successful communicative exchanges need to be cooperative in the first place and how cooperation should be characterized exactly. Game-theoretic evolutionary models of communication show that deception does not lead communication to break down (Zollman et al., 2013). In general, this work indicates that all animals deceive to some extent but humans are special in that their communication system is combinatorial. (Human) combinatorial communication systems are more efficient and they allow for a particular kind of deception where speakers occasionally use atypical messages to deceive (Lachmann & Bergstrom, 2004). Thus, humans may deceive in a particular way that other animals do not.

Inspired by such evolutionary approaches, Pinker et al. (2008) developed a model of indirect speech that explicitly incorporates a mixture of cooperation and conflict. Pinker et al. (2008) show that in situations of conflict, speakers prefer to implicate rather than to communicate information explicitly. Their model predicts that it is optimal to communicate indirectly in situations of conflict, as it allows the speaker a loophole to plausibly deny the implicated meaning if openly challenged. For example, a speaker may say *Your shirt is not pretty* to implicate that ‘the shirt is ugly’. The utterance *The shirt is ugly* would be more efficient to convey this meaning but it threatens the hearer’s face (Brown & Levinson, 1987). Hence, implicating in such cases may be motivated by a different payoff (one not based on efficiency considerations); rather, it allows speakers to pursue their own goals while managing their social relationships (see Gotzner & Scontras, 2024; Pinker et al., 2008). At the same time, the potential for deception and miscommunication might be greater when content is implicated rather than explicitly communicated. Implicated content might not be immediately targeted by discourse participants. Further, if hearers do target it, the speaker can plausibly deny the implicated content (Gotzner & Scontras, 2024; Pinker et al., 2008). Overall, indirect language leaves many interpretative options and it depends on the trust and social relationship of interlocutors, which inferences are derived.

These game-theoretic approaches diverge from the Gricean model as they explicitly model (a mixture of cooperation and) conflict in the communication system. Speakers are naturally taken to be strategic and to seek plausible deniability (Lee & Pinker, 2010). Strictly Gricean models, in turn, would assume that people follow the cooperative principle and that the hearer is filling in propositions that are necessary to preserve the assumption that the speaker is trying to be informative, truthful, relevant and clear. Listeners are predicted to run the same inferential mechanism even for non-cooperative speakers but they may cancel the implicated meaning after having derived it (see for example Goodman & Stuhlmüller, 2013 and the discussion in the Scalar Implicature as a Test Case section below).

### Political Speech

Political speech is a particularly interesting test case for several reasons. First, cooperation cannot be a precondition for political communication, as it is clear to everyone that politicians pursue their own strategic goals. Second, politicians often leave themselves a loophole for argumentative effectiveness and to conceal questionable pieces of information (Lombardi Vallauri, 2016; Mannaioli, 2025). Third, politicians are usually not addressing a single individual but a mixed crowd, which includes subgroups having different beliefs. Specific communicative strategies allow them to maintain a public image while addressing their fellow supporters, thus maintaining plausible deniability (see for example Henderson & McCready, 2024 and Beaver & Stanley, 2023 for work on dog whistles, a common type of coded language in political speech). For these reasons, political speech is an ideal test case to investigate whether participants assume the cooperative principle and derive implicated content even for speakers that are not fully cooperative.

Politicians also portray specific ideologies, which is reflected in their language choice. A recent study by Wicke and Bolognesi (2024) used large language models to compare the political speeches of Donald Trump and Kamala Harris. The results revealed distinct linguistic patterns associated with the framing of conservative ideologies for Trump and liberal ones for Harris. For example, while Harris was using singular (*I*) and plural pronouns (*we*) equally, Trump predominately used singular pronouns.

To understand the impact of political language on hearers, research on social categorization and politically-motivated reasoning is particularly relevant. Seminal work in social psychology by Tajfel et al. (1971) showed that people show favorable attitudes and behavior towards ingroup members while being disfavorable to outgroup members. Language plays an important role in promoting stereotypes: the same desirable behavior is encoded at a higher level of abstraction when performed by an ingroup member, inducing a generalization to character traits (Maass et al., 1989). In contrast, the behavior of outgroup members is just encoded as a concrete instance (ibid.). Group membership affects a range of communicative phenomena including pragmatic reasoning, for example jokes are appreciated more when participants are a member of the speaker’s ingroup (Morisseau et al., 2017). Such effects can be mediated by disaffiliation with the outgroup (ibid.), and participants with low theory of mind abilities have a harder time arriving at a pragmatic interpretation for outgroup members (Kuperwasser & Shetreet, 2025). What is more, Kahan (2013) argued that political content promotes a particular kind of tribal reasoning. This account essentially assumes that people negotiate identities when it comes to politics and are therefore particularly prone to have cognitive biases (see also Kahan, 2015; Seremeta et al., 2024). This can have the effect that one evaluates the content less deliberately if it is consistent with the prior belief of the group one affiliates with. Sperber et al. (2010) make a related point regarding epistemic authorities such as religious leaders and gurus: When the source gains an inflated reputation, people defer more to the source rather than checking the content promoted by the source deliberately. In such cases, people optimize agreement and therefore assume a charitable interpretation even when the stated information is clearly inconsistent with their prior beliefs (for example, regarding claims about Mary being a virgin when she gave birth to Jesus).

### Scalar Implicature as a Test Case

The current study uses a particular kind of implicature—scalar implicature—as a test for the role of cooperation in communicated content. Scalar implicatures have become one key test case for theoretical debates and the experimental turn in pragmatics (see Noveck, 2018 for a historical overview). On the one hand, scalar implicatures are a good test case because they are generalized implicatures and the expressions they involve stand in a paradigmatic relationship (e.g, the informationally weak quantifier *some* and the stronger *all*, see Horn, 1972 who introduced the notion of scales). On the other hand, scalar implicatures have been subject to the most controversial debate regarding the mechanisms underpinning these inferences. While many more recent accounts inspired by Grice maintain a role of speaker meaning and epistemic assessment in scalar implicatures (including Neo-Gricean accounts by Horn, 1972; Sauerland, 2004; relevance-theoretic accounts such as Sperber & Wilson, 1986 and game-theoretic accounts like Asher & Lascarides, 2013), other scholars have proposed to embed such inferences within compositional grammar (e.g., Chierchia, 2004; see Gotzner & Romoli, 2022 for an overview of current debates).

Crucially, empirical studies show that participants need to invest processing resources for computing scalar implicatures (Bott & Noveck, 2004) as they reason about the speaker’s epistemic state and their intentions (e.g., Breheny et al., 2013; Ronderos & Noveck, 2023). When participants interact with an outgroup member, they more often opt for a literal interpretation, especially individuals with lower theory of mind abilities (Kuperwasser & Shetreet, 2025). Studies of this kind suggest that participants need to do some mind reading to arrive at a pragmatic interpretation. Importantly though, implicatures seem to be understood even if interlocutors are not being fully cooperative, for example in competitive games and adversarial court room scenarios (Asher & Lascarides, 2013; Franke et al., 2020). Further, Goodman and Stuhlmüller (2013) show that scalar implicatures are canceled if it is common knowledge that the speaker only has partial information (see also Porrini et al., 2025). Their Rational Speech act model implements a Gricean communication system and predicts that scalar implicatures would still be generated even if the speaker is not fully cooperative. The lack of full knowledge would have the effect that implicatures are canceled after being generated. In a similar vein, Mazzarella et al. (2018) found that participants are less likely to accept scalar implicatures as true in face-threatening contexts, for example in sentences like “Some people hated your speech” (while generating them to an equal extent in face-threatening and face-boosting contexts). In sum, previous work indicates that epistemic factors related to the speaker play a critical role in the inference process, either leading participants to not generate scalar implicatures in the first place or to cancel them later on.

The experimental literature further shows a large amount of variability in the extent to which participants derive scalar implicatures across different expressions (Doran et al., 2009; Gotzner et al., 2018a, 2018b; Van Tiel et al., 2016). For example, while vague expressions like *attractive* rarely trigger scalar implicatures, other expressions that involve fixed upper bounds (*certain*) are more robust triggers (see examples 1 and 2 in the Methods section). This variability can to a large extent be accounted for with semantic factors in the case of adjective scales (Gotzner et al., 2018a, 2018b). For example, the threshold for what counts as (*attractive*) is itself context-dependent (Kennedy & McNally, 2005) and for that reason it is less likely that participants will judge this term to exclude a stronger term like *stunning* (especially if it does not denote an endpoint). What is more, vague and subjective adjectives are prone to be used for face-saving reasons (Terkourafi et al., 2020), as the speaker can plausibly deny the implicated meaning and thus navigate a situation of conflict (Gotzner & Scontras, 2024).

The current study uses a manipulation of speaker (political candidate) and voter group as an index of the role of cooperativity in endorsing scalar implicatures. Between-item variation is used as an index of whether participants take into account the semantic meaning of the words candidates use and what role social aspects such as face-related concerns might play. Given the above discussion, one expects that scalar implicatures are more readily generated when the participant trusts the speaker and assumes that they are being cooperative. Thus, we expect both variation between participants and between different expressions regarding the likelihood with which participants compute scalar implicatures (see also Hu et al., 2022).

## CURRENT STUDY

### Design and Hypothesis

The current study used a 2 speaker (Harris vs. Trump) × 2 voter group (Democratic vs. Republican voters) design. Participants were recruited via Prolific with pre-screeners on political affiliation, their native language English and U.S. Nationality and residence. Participants read short statements uttered by Harris or Trump and were asked to judge whether they would endorse a stronger statement involving a scalar implicature. The preregistered hypothesis is that more scalar implicatures will be derived when voter’s beliefs align with those of the speaker (https://doi.org/10.17605/osf.io/6284d). This hypothesis predicts an interaction of voter group and speaker. The experiment was run on 1st November 2024, when according to the National polls, each candidate had an equal chance of winning the election.^1^

### Methods

#### Participants.

The experiment was administered to 120 participants on Prolific with pre-screeners for the age range (18 and 67), with English as native language, the United States as nationality and country of residence, along with the participant’s self-declared political affiliation (Democratic or Republican). In the Democratic group, 45 women and 16 men with a mean age of 38.61 (*SD* 11.27) participated. In the Republican group, 36 women and 26 men with a mean age of 40.92 (*SD* 11.07) participated in the experiment. Participants had responded to the pre-screening questions at a previous point in time, thus no attention was drawn to the fact that they were recruited based on their political affiliation. The experiment was hosted on the experiment server L-REX (Starschenko & Wierzba, 2025).

#### Task and Items.

**Figure 1.**
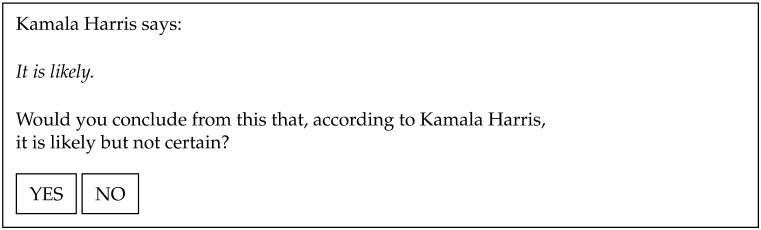
Example trial: upper bound scale, speaker Harris.

**Figure 2.**
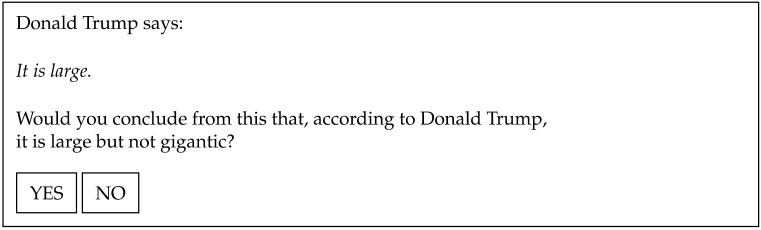
Example trial: extreme adjective, speaker Trump.

The list of items included 70 adjective pairs with weak and strong scale-mates, taken from a study by Gotzner et al. (2018b). For example, *likely* is informationally weaker than *certain*. These adjectives were pretested for a number of semantic factors (see the supplementary materials of Gotzner et al., 2018b for a list of items and their characteristics.). The weak term of each pair was embedded in a statement that was said to be uttered by Kamala Harris or Donald Trump and it was the participant’s task to judge whether or not a scalar implicature was triggered by a weak statement. For example, participants judged whether *likely* should be taken to mean ‘likely but not certain’, according to the speaker.^2^ Figure 1 shows an example trial with Harris as the speaker and a scale that includes a bounded strong term (*certain*), which denotes an endpoint on the relevant scale. This is typically an item that prompts high proportions of YES responses in scalar implicature tasks (see for example Gotzner et al., 2018b; Van Tiel et al., 2016). Figure 2 shows an example trial with Trump as the speaker and a scale that includes an extreme strong term (*gigantic*), which denotes a high degree but not an endpoint. This is typically an item that gets low rates of scalar implicatures.

### Results

#### Main Analysis of Speaker and Voter Group.

**Figure 3.**
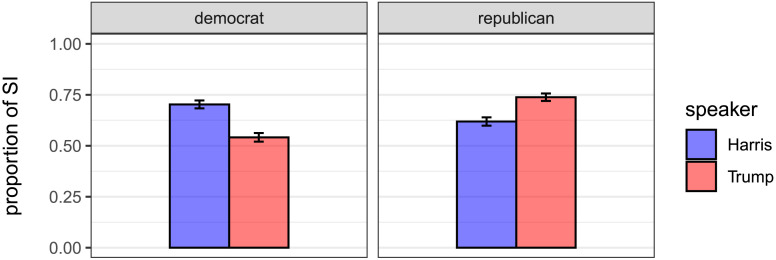
Proportions of scalar implicatures for Harris and Trump in Democratic and Republican voters. Error bars indicate 95% confidence intervals.

Figure 3 displays the proportion of scalar implicature among Democratic and Republican voters for the speaker Harris and Trump, respectively. To test the pre-registered hypothesis, judgments were analyzed with logit mixed-effects model predicting scalar implicature as a binary variable. The model included the sum-coded factors speaker (Harris vs. Trump), voter group (Democratic vs. Republican), an interaction of the two factors, together with the maximal random effects structure justified by the design (see Table 1). The model showed an interaction between voter group and speaker (*β* = −0.45, *z* = −5.04, *p* < 0.0001). There were no main differences between voter groups and no main differences between the two candidates. The attested interaction effect confirms the pre-registered hypothesis: participants derive more scalar implicatures when their own beliefs align with the affiliation of the candidate.

**Table 1.** Logit mixed effects model: glmer(SI ∼ speaker * voter) + (1 + speaker|item) + (1 + speaker|participant).

	Variance	*SD*	Corr	
Random effects:				
participant (intercept)	2.12492	1.4577		
speaker	0.87526	0.9356	−0.15	
item (intercept)	0.53909	0.7342		
speaker	0.0298	0.1727	−0.21	
	Estimate	*SE*	*z*-value	*p*-value
Coefficients:				
(Intercept)	0.96272	0.16001	6.017	0.0001
speaker	−0.11850	0.09231	−1.284	0.199
voter	−0.16929	0.13346	−1.269	0.205
speaker:voter	−0.45134	0.08954	−5.040	0.0001

All results and analysis scripts, together with the preregistered hypothesis, can be found on this OSF repository: osf.io/v98dt.

### Group Analysis

**Figure A4.**
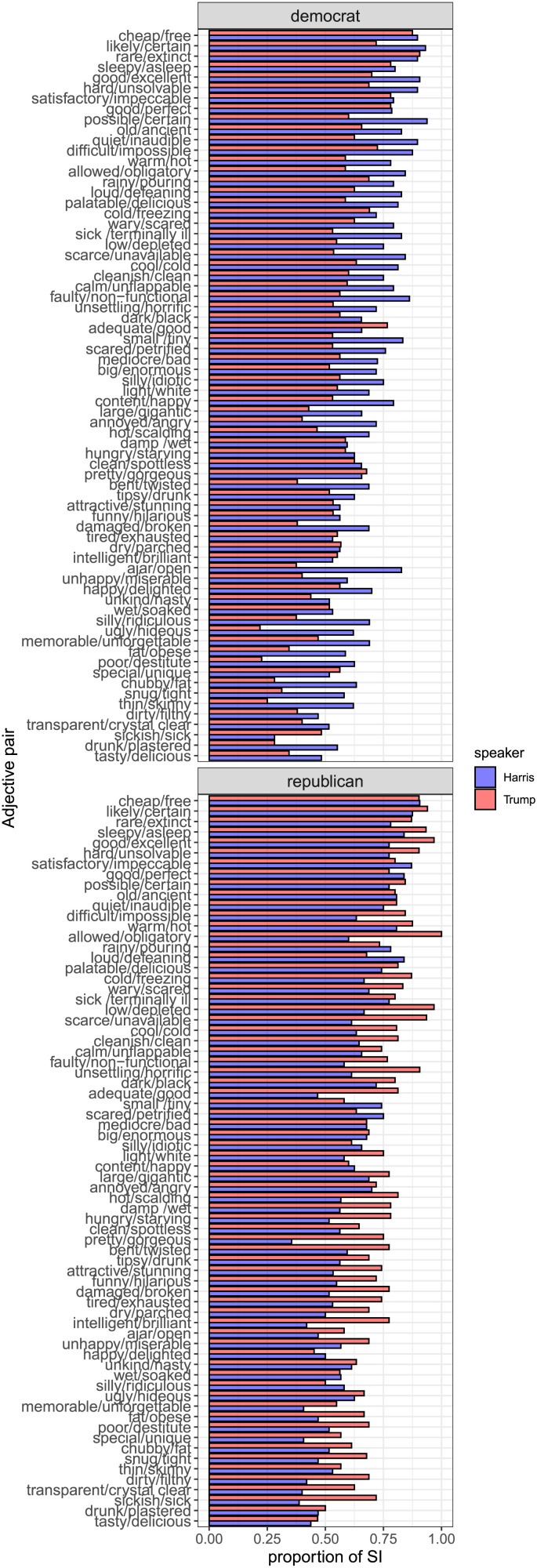
Proportions of scalar implicatures by adjective.

In order to assess whether participants were more likely to derive scalar implicatures with political candidates, we can compare the current data to the base rates of the original study by Gotzner et al. (2018b). In this study, the items were uttered by two people with common names (John or Mary) and all sentences were the same as in the current study. In this original setup, participants arguably did not construe a real personae and did not reason about the speaker potentially being non-cooperative or an outgroup member. A further question that can be addressed with such an analysis is whether boosted inference rates are due to affiliation with ingroup members or rather disaffiliation with outgroup members (see for example Kuperwasser & Shetreet, 2025; Morisseau et al., 2017).^3^ For this purpose, an average score by adjective for ingroups (Democratic voters - Harris, Republican voters - Trump) and outgroups (Democratic voters - Trump, Republican voters - Harris) was computed. Figure A4 in the Appendix shows the mean proportion of scalar implicatures across groups. These data were submitted to three two sample *t*-tests with paired values across adjectives. The alpha level was adjusted for multiple comparison (Bonferroni corrected alpha level 0.015). There was a significant positive difference for ingroups relative to the baseline condition (*M*_diff_ = 0.34, *t*(69) = 20.09, *p* < .001; 95% CI [0.3018, 0.3684]) as well as a positive effect for outgroups compared to the baseline (*M*_diff_ = 0.19, *t*(69) = 10.78, *p* < .001; 95% CI [0.1586, 0.2306]). These analyses indicate that participants were on average more likely to derive scalar implicatures for political candidates relative to a baseline with common names (no political candidates). The final *t*-test compared ingroup and outgroup ratings directly. This test showed significantly higher ingroup ratings compared to outgroup ratings (*M*_diff_ = 0.14, *t*(69) = 14.63, *p* < .001; 95% CI [0.1213, 0.1596]), indicating that communicated content is understood best among ingroup members.

### Semantic Factors

Previous work has indicated that item-based variability for adjectival expressions is to a large extent accounted for by semantic factors (Gotzner et al., 2018b). To assess the role of semantic factors, additional models were fit crossing the factors boundedness and adjectival extremeness with speaker and voter group. In previous work, scalar implicature has been found to be higher for scales that involve a bounded stronger term such as *certain* compared to non-bounded stronger terms *hot* (Gotzner et al., 2018b; Van Tiel et al., 2016). If the pairs of expressions involve an extreme strong term like *gigantic*, scalar implicature rates tend to be lower (cf. Figure 2).

The Appendix provides additional figures and model results for the factors boundedness and extremeness across conditions as well as individual item variability. The full logit mixed effects including an interaction with boundedness revealed a higher proportion of scalar implicature for bounded terms compared to non-bounded terms (*β* = 0.32, *z* = 3.6, *p* < 0.0001). There was a significant three-way interaction of voter group and boundedness such that boundedness had a stronger effect on Democratic voters’ judgments (*β* = 0.06, *z* = 2.12, *p* < 0.05). In addition, there was a significant three-way interaction of speaker, voter group and boundedness with boundedness mattering less in Republican voter’s judgments especially for statement by Harris (*β* = −0.06, *z* = −2.08, *p* < 0.05). A second model with extremeness as a factor showed that scalar implicatures for extreme terms were derived less compared to non-extreme terms (*β* = −0.22, *z* = −2.33, *p* < 0.05). Moreover, there was a significant three-way interaction of speaker, voter group and extremeness with a smaller effect of extremeness in Republican voter’s judgments, especially for statements by Harris (*β* = 0.07, *z* = 2.47, *p* < 0.05). In both models, the critical interaction between speaker and voter persisted. More information on individual item variability can be found in the OSF repository: osf.io/v98dt.^4^

Overall, these analyses of item variability indicate that semantic factors are less predictive among Republican voter groups and partly more so when they concern the opposing candidate, Kamala Harris. These results add to a growing literature indicating that scalar implicature derivation is subject to item-based as well as participant-related variability (see for example Hu et al., 2022 and subsequent work).

## DISCUSSION

The current study used underinformative statements ostensibly uttered by political candidates to probe the role of belief alignment for interpreting communicated content. To assess how people calibrate cooperation and the level of trust they grant the speaker, Republican and voters were tested on how they interpret statements made by Trump and Harris. The experiment was run before the U.S. presidential election on November 1st 2024, when it was not clear which candidate will win. The results confirmed the main hypothesis that scalar implicatures are more likely to be derived when beliefs between voter and candidate align. Furthermore, the results showed that the proportion of scalar implicatures is higher on average for both ingroup and outgroup membership compared to baseline speakers with common names (i.e., where there is no manipulation of group membership or political affiliation).

The main assumption in Sperber et al. (2010)’s work is that hearers take into account the competence of the speaker and their benevolence in calibrating their level of trust towards the speaker. Ultimately, such epistemic assessments are crucial for selecting cooperative partners more generally. Previous work has shown that speaker competence affects interpretation (Pozzi & Mazzarella, 2024) as well as the social identity of the speaker (for example female speakers are taken to communicate more indirectly Gotzner & Mazzarella, 2021; see also Beltrama & Schwarz, 2024 for other aspects of personality influencing interpretation). Furthermore, the derivation of pragmatic inferences depends on speaker intentionality (Breheny et al., 2013; Ronderos & Noveck, 2023) and group membership (Kuperwasser & Shetreet, 2025; Morisseau et al., 2017). In the current study, the speaker that aligns with a participant’s political beliefs is likely to be considered more trustworthy. The effects cannot be due to a mere form of speaker competence in the sense of the level of confidence the individual candidates portray (see Pozzi & Mazzarella, 2024 for discussion on different notions of speaker competence and trust). If that were the case, we would expect to see main effects of speaker type rather than an interaction of speaker and voter group. The current work, in turn, does not provide evidence for main differences in how statements ascribed to Donald Trump and Kamala Harris are interpreted. Instead, judgments depend on whether the speaker aligns with the political beliefs of the voter group. The baseline difference from a speaker with a common name indicates that participants readily compute the implicated content for statements by political candidates. This is reminiscent of power asymmetries influencing indirect language use and interpretation—where listeners take high status speakers to intentionally implicate content (Brown & Levinson, 1987; Lee & Pinker, 2010). The current study showed that belief alignment further boosts the likelihood that an inference is being made above and beyond the high power status attributed to politicians.

This main result indicates that individuals may arrive at different implicated content from the exact same statement, depending on who the speaker is and what their own beliefs are. A previous EEG study by Van Berkum et al. (2009) indicated that the brain rapidly unlocks value-based disagreement when participants read statements that explicitly violate their political/moral beliefs (e.g., *I think that euthanasia is an acceptable course of action*). The current study looked at implicatures, which are even more susceptible to misleading uses and miscommunication than explicit content. What is left open from the current work is at which processing stage the effect of speaker-voter alignment occurs. It could be that participants integrate their trust towards the speaker during incremental language comprehension or later in their acceptance of the implicated information, as has been suggested by Sperber et al. (2010) and Mazzarella et al. (2018). Although we do not have processing evidence for teasing apart these possibilities, the current findings remain profound. They suggest that individuals belonging to different groups may adjust their interpretation of language, depending on the trust they grant the speaker.

There is some evidence for a difference between groups from the current results in that Republican voters showed a lower sensitivity to semantic factors compared to Democratic voters. This was the case overall and even more so for the opposing candidate Harris. These effects indicate that participants do not simply endorse anything political leaders say but they do process the basic meaning of expressions. The lower sensitivity to semantic factors in Republican voters might stem from well-known ingroup and outgroup biases (Tajfel et al., 1971). On the one hand, Republicans might affiliate more strongly with Trump (than the extent to which Democratic voters affiliated with Harris before the election). On the other hand, Republicans might judge the content of the opposing candidate less deliberately. The additional group analysis indicated that ingroups showed the greatest proportion of scalar implicatures. Furthermore, even outgroup members showed a boost relative to the baseline speaker. These findings suggest that the results are mostly driven by better understanding of implicated content as a function of group membership (rather than derogation or pragmatic impairment for outgroup members). While previous work found evidence that outgroup members compute more literal responses compared to baseline confederate speakers (Kuperwasser & Shetreet, 2025), the current study found that on average participants more strongly endorse the implicated content of famous politicians, likely due to their position of power. Thus, participants seem to grant a stance of trust to political candidates and understand implicatures; but they may cancel implicatures in the face of opposing political views. Overall, the current results support earlier findings that scalar implicatures are understood even when interlocutors are not fully cooperative (Asher & Lascarides, 2013; Franke et al., 2020).

Information within a group often spreads from a single source and therefore the trustworthiness of the original source is the key factor for whether information is accepted and less so the evidence and arguments for its content (Sperber et al., 2010). Furthermore, the members of a community are biased to adopt the majority opinion (Henrich & Boyd, 1998). As a consequence, epistemic authorities like religious leaders may get inflated reputations and people can no longer question what they say without rejecting the authority itself (Sperber et al., 2010). Political communication could be subject to a similar mechanism, especially when propaganda tactics promoting polarization and avoidance of fact-checking are in place (see also Bergstrom & West, 2021 for discussion). In general, political language is particularly susceptible to social dynamics. When it comes to politics, ideologically-motivated reasoning promotes the interest of an individual to signify their loyalty to a group, thus leading to cognitive biases (see Kahan, 2013). Overall, the current work indicates is that communication and information processing in groups is highly socially-motivated.

## CONCLUSION

Recent years have seen a social turn in the study on meaning, where the relation between linguistic meaning, ideologies and the social world is being integrated and modeled with the same tools of formal semantics and game theory (see for example Burnett, 2023 and Henderson & McCready, 2024). The current study argues for a similar shift in studies on language and communication. Through language, humans not only exchange information implicitly or explicitly but, at the same time, they construct social identities and relationships. Where does this leave us regarding the role of cooperation in communication, as assumed in the Gricean framework? The results suggest an important qualification: Rather than being a precondition for successful communication, cooperation is calibrated in social groups and beliefs affect the level of trust the speaker is being granted. As a consequence, groups with different political predispositions may interpret the same statement differently, depending on which political candidate uses it. More specifically, the results suggest that even in the face of opposing views, hearers understand the implicated content of a statement but they may not accept it.

## ACKNOWLEDGMENTS

I would like to thank Heather Burnett and Elli Tourtouri for insightful comments on this work and Radim Lacina for implementing the items.

## FUNDING INFORMATION

This research was supported by the DFG (Emmy Noether grant awarded to NG, project number 441607011).

## DATA AVAILABILITY STATEMENT

All experimental materials, data and analysis scripts are available on the following OSF repository: osf.io/v98dt.

## Notes

^1^ https://projects.fivethirtyeight.com/polls/president-general/2024/national/.^2^ In this version of the task, negative strengthening is blocked since the literal meaning is taken together with the scalar implicature (‘weak but not strong’) (see Gotzner et al., 2018a).^3^ I would like to thank the reviewers of this manuscript for bringing up this further literature and suggestions for additional analyses that were not preregistered.^4^ Another exploratory analysis suggested by an anonymous reviewer revealed that the proportion of scalar implicature was higher when the test sentence included the pronoun *it* and when the pronoun matched in gender with the speaker (*she* for Harris and *he* for Trump). Further research directly manipulating theses aspects would be required to draw firm conclusions about their relevance.

## Figures and Tables

**Figure A1.**
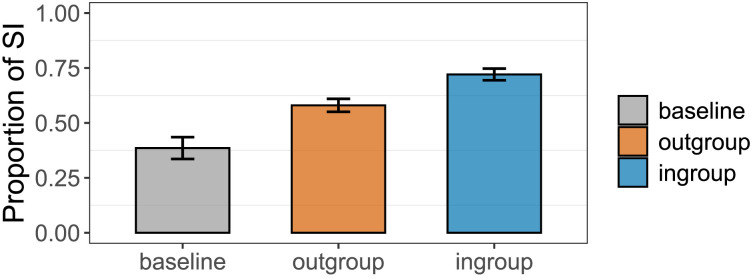
Proportions of scalar implicatures for baseline speaker with common name (from Gotzner et al., 2018b), ingroups (averaging Trump - Republican and Harris - Democratic conditions) and outgroups (averaging Trump - Democratic and Harris - Republican conditions). Error bars indicate 95% confidence intervals.

**Figure A2.**
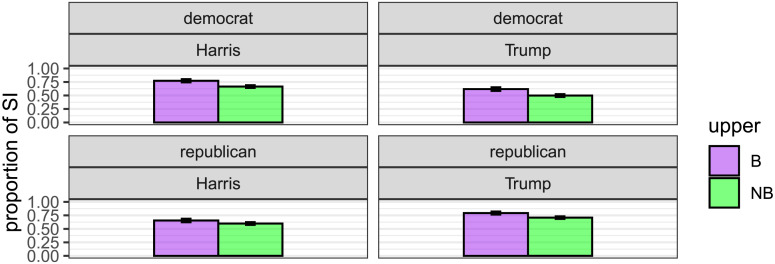
Proportions of scalar implicatures for bounded B (e.g., *certain*) and non-bounded NB strong terms (e.g., *good*), the speaker Harris and Trump in Democratic and Republican voters. Error bars indicate 95% confidence intervals.

**Figure A3.**
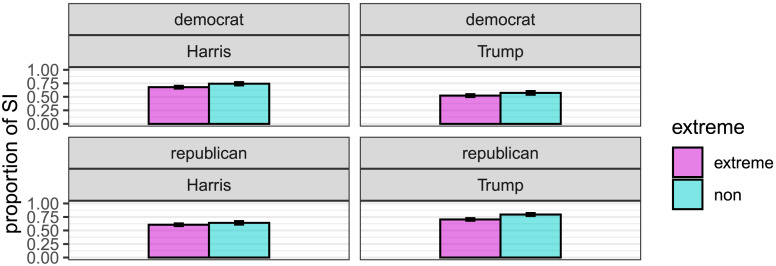
Proportions of scalar implicatures for extreme (e.g., *gigantic*) and non-extreme strong terms (e.g., *good*), broken by the speaker Harris and Trump in Democratic and Republican voters. Error bars indicate 95% confidence intervals.

## APPENDIX

**Table A1.** Logit mixed effects model: glmer(SI ∼ speaker * voter * boundedness) + (1 + speaker|item) + (1 + speaker|participant).

	Variance	*SD*	Corr	
Random effects:				
participant (intercept)	2.13411	1.4609		
speaker	0.87376	0.9348	−0.15	
item (intercept)	0.45147	0.6719		
speaker	0.02677	0.1636	−0.38	
	Estimate	*SE*	*z*-value	*p*-value
Coefficients:				
(Intercept)	1.05761	0.15856	6.670	2.56e−11
speaker	−0.11029	0.09295	−1.187	0.235396
voter	−0.14527	0.13420	−1.082	0.279038
upper	0.32302	0.08963	3.604	0.000313
speaker:voter	−0.47479	0.09018	−5.265	0.0001
speaker:upper	0.04159	0.03671	1.133	0.257217
voter:upper	0.06364	0.03010	2.115	0.034466
speaker:voter:upper	0.06266	0.03014	−2.079	0.037638

**Table A2.** Logit mixed effects model: glmer(SI ∼ speaker * voter * extremeness) + (1 + speaker|item) + (1 + speaker|participant).

	Variance	*SD*	Corr	
Random effects:				
participant (intercept)	2.12532	1.4578		
speaker	0.87602	0.9360	−0.16	
item (intercept)	0.49922	0.7066		
speaker	0.02966	0.1722	−0.24	
	Estimate	*SE*	*z*-value	*p*-value
Coefficients:				
(Intercept)	1.020548	0.160116	6.374	0.0001
speaker	−0.112153	0.093008	−1.206	0.2279
voter	−0.172717	0.133799	−1.291	0.1967
extremeness	−0.215357	0.092367	−2.332	0.0197
speaker:voter	−0.473784	0.090067	−5.260	0.0001
speaker:extremeness	−0.019501	0.036425	−0.535	0.5924
voter:extremeness	0.009396	0.029300	0.321	0.7484
speaker:voter:extremeness	0.072510	0.029342	2.471	0.0135
